# A review on the role of NDRG1 in different cancers

**DOI:** 10.1007/s11033-023-08540-z

**Published:** 2023-05-30

**Authors:** Soudeh Ghafouri-Fard, Sara Ahmadi Teshnizi, Bashdar Mahmud Hussen, Mohammad Taheri, Guive Sharifi

**Affiliations:** 1 Department of Medical Genetics, School of Medicine, Shahid Beheshti University of Medical Science, Tehran, Iran; 2 Phytochemistry Research Center, Shahid Beheshti University of Medical Science, Tehran, Iran; 3 Department of Clinical Analysis, College of Pharmacy, Hawler Medical University, Kurdistan Region, Erbil, Iraq; 4 Institute of Human Genetics, Jena University Hospital, Jena, Germany; 5 Urology and Nephrology Research Center, Shahid Beheshti University of Medical Science, Tehran, Iran; 6 Skull Base Research Center, Loghman Hakim Hospital, Shahid Beheshti University of Medical Science, Tehran, Iran

**Keywords:** NDRG1, Cancer, Expression, Carcinogenesis, Biomarker

## Abstract

NDRG1 is a member of the α/β hydrolase superfamily that resides in the cytoplasm and participates in the stress responses, hormone response, cell growth, and differentiation. Several studies have pointed to the importance of NDRG1 in the carcinogenesis. This gene has been found to be up-regulated in an array of cancer types such as bladder, esophageal squamous cell carcinoma, endometrial, lung and liver cancers, but being down-regulated in other types of cancers such as colorectal, gastric and ovarian cancers. The current study summarizes the evidence on the role of NDRG1 in the carcinogenic processes in different types of tissues.

## Introduction

**Fig. 1:**
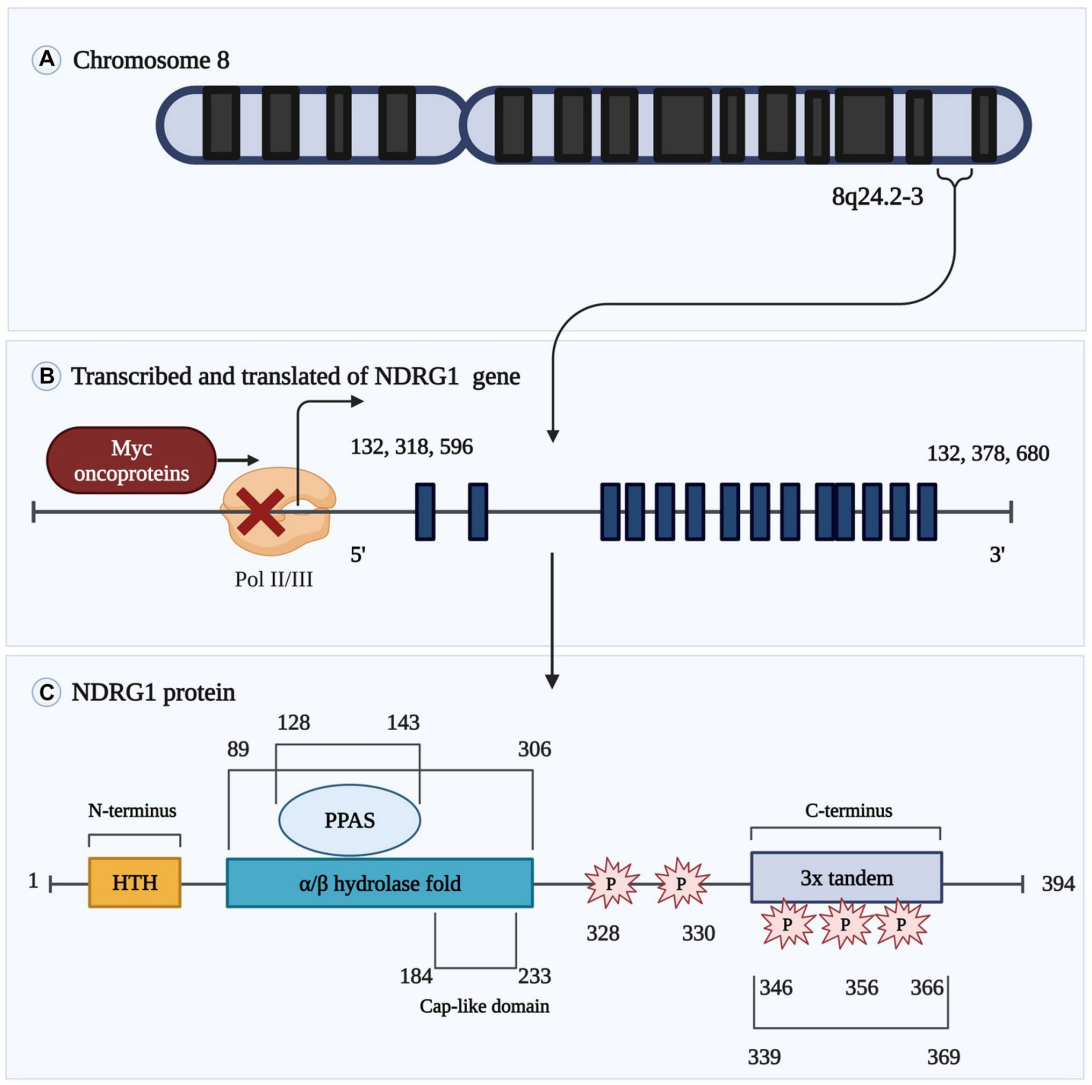
A graphical illustration of the component parts that make up the modular framework of NDRG1

N-myc downstream regulated 1 (NDRG1) is encoded by a gene located on chromosome 8q24.22, roughly 60 kb in size. This gene provides instructions for making a protein of 43 kDa (made up of 394 amino acids) that is extremely stable and is highly conserved among multicellular creatures. The mRNA for this gene is 3.0 kb in size [1] (Fig. 1). The *NDRG1* gene is a part of the human *NDRG* family, which also includes the *NDRG2*, *NDRG3*, and *NDRG4* genes. These genes are only 53–64% similar to one another [2]. As a member of the α/β hydrolase superfamily, this cytoplasmic protein takes part in the regulation of stress response, hormone response, cell growth, and differentiation. In addition, it has a crucial role in p53-mediated activation of caspase and apoptosis. Mutations in *NDRG1* gene have been shown to cause Charcot-Marie-Tooth disease type 4D [3]. In addition to the cytoplasm, this protein can be found in the cell membrane and nucleus of the cells.

More recently, studies have pointed to the importance of NDRG1 in the carcinogenesis. This gene has been found to be up-regulated in an array of cancer types, but being down-regulated in other types of cancers. The current study summarizes the evidence on the role of NDRG1 in the carcinogenic processes in different types of tissues. We have categorized this paper into distinct subtitles based on the obtained evidence from cell lines, xenograft models of cancer and expression assays in clinical samples.

## Role of NDRG1 in cancer based on cell line studies

Cell line studies in triple-negative breast cancer (TNBC) have shown important effect of HJURP/YAP1/NDRG1 axis in these cells. Expression of HJURP has been found to be up-regulated in TNBC compared to other subtypes of breast cancer. This evolutionarily conserved chaperone can influence ubiquitination modification level of YAP1 protein, thus regulating its downstream transcriptional activities. YAP1 can induce transcription of NDRG1 through binding to its promoter. The HJURP/YAP1/NDRG1 axis can influence proliferation and chemosensitivity of TNBC cells [4].

Another study has re-analyzed the transcriptomic data from TNBC cybrids with a number of nuclear or mitochondrial donor cells. This study has shown up-regulation of 149 genes in the cybrids with mitochondria, among them being NDRG1. NDRG1 has been shown to be co-located with PVT1, and EXT1 on 8q24, a region that harbors amplification in breast cancer. NDRG1 has exhibited the most significant under-expression in the cybrids with benign mitochondria. NDRG1 silencing has led to reduction of proliferation of SUM-159 TNBC cells. Taken together, this study has demonstrated the role of mitochondria in facilitating over-expression of NDRG1 in TNBC [5].

Moreover, another study in breast cancer cells has shown that NDRG1 silencing leads to reduction of cell proliferation rate, abnormalities in lipid metabolism such as enhancement of incorporation of fatty acids into neutral lipids and lipid droplets. On the contrary, NDRG1 expression has diminished lipid droplet construction under nutrient supplied and starvation circumstances [6].

Besides, expression of NDRG1 can be affected by hormones. For instance, a cell line study has shown up-regulation of SGK1 and NDRG1 following treatment with progesterone. These observations have been accompanied by down-regulation of two miRNAs that target SGK1 3'UTR, namely miR-29a and miR-101–1. Authors have also demonstrated progesterone-mediated transcriptional and post-transcriptional control of SGK1 expression, resulting in over-expression of NDRG1 by a number of genes whose expressions are controlled by the transcription factor AP-1. NDRG1 can reduce activity of some kinases, thus reducing invasion and migratory potential of breast cancer cells [7].

In addition to hormones, expression of NDRG1 has been shown to be regulated by the long non-coding RNA (lncRNA) NDRG1 overlapping transcript 1 (NDRG1-OT1). Expression of this lncRNA is induced under hypoxic conditions. Notably, NDRG1-OT1 can inhibit expression of NDRG1 at both transcript and protein levels. NDRG1-OT1 enhances degradation of NDRG1 through ubiquitin-mediated proteolysis. Moreover, NDRG1-OT1 can decrease NDRG1 promoter activity. Most notably, different segments of NDRG1-OT1 have been shown to exert opposite impacts on NDRG1. While the first quarter segment of this lncRNA has no impact on the promoter of NDRG1, the second and third quarter segments represses and improves NDRG1 promoter activity, respectively. Finally, the fourth quarter segment of this lncRNA decreases activity of this promoter through reducing KHSRP under hypoxia [8].

5-Aza-2′-deoxycytidine is another agent that induces expression of NDRG1. While NDRG1 is expressed in the MDA-MB-231, this gene is expressed in T47D cells only following treatment with 5-Aza-2′-deoxycytidine. This observation shows the impact of DNA methylation on NDRG1 expression. NDRG1 gene promoter has a high level of methylation in T47D cells in spite of low level of methylation of this promoter in MDA-MB-231 cells [9].

**Fig. 2:**
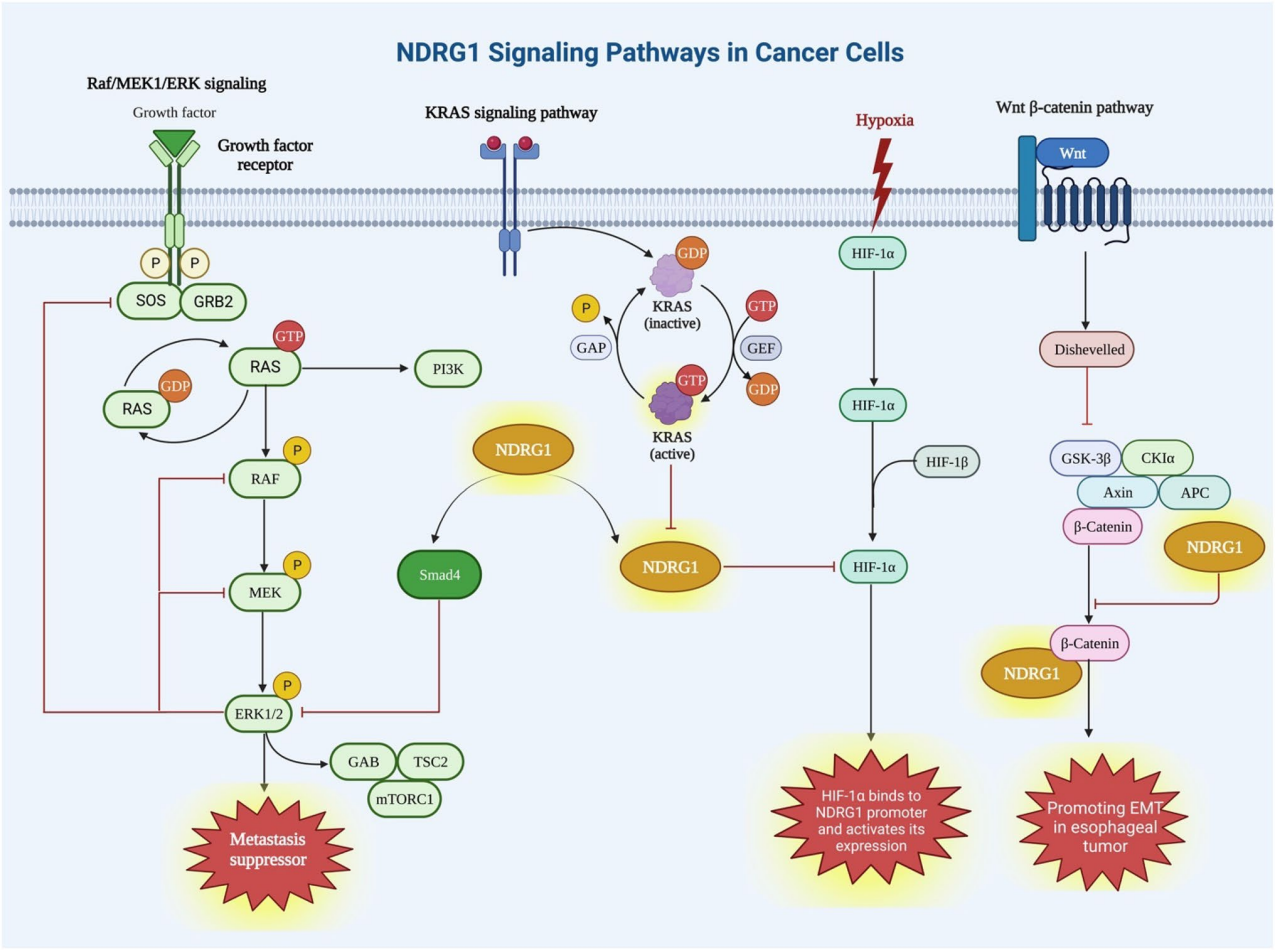
An illustration of the individual components of the architecture of NDRG1 signaling pathways in cancer cells

Furthermore, by modulating TLE2 and β-catenin expression, NDRG1 regulates Wnt pathway activation and EMT in esophageal cancer cells [10]. This suggests that NDRG1 in esophageal cancer cells plays a pro-oncogenic role through modulating tumor development (Fig. 2).

Additionally, K-Ras is critical for controlling in vitro NDRG1 protein level in pancreatic ductal adenocarcinoma (PDAC) cancer cells through ERK signaling [11]. Notably, NDRG1 expression reduces the amount and activity of HIF1 by inhibiting K-Ras downstream Akt/mTOR signaling. In particular, NDRG1 plays an especial part in controlling PDAC cancer metabolism. NDRG1 mediates downregulation of many glycolysis essential enzymes, including GLUT1, HK2, LDHA, and PDK1 [11]. Furthermore, it appears that NDRG1 inhibits glycolysis enzymes via decreasing the activity of HIF1, which may be the cause of this phenomenon.

Moreover, NDRG1 communicates with its target cells and other cells via signaling pathways and molecular motors. For instance, NDRG1 is associated with cancer metastasis via its function in Raf/MEK1/ERK signaling. Ras is bound to GTP upon activation of GFRs [12]. The downstream targets are activated when the active Ras:GTP phosphorylates Raf/MEK1/ERK1/2. Smad4 is upregulated by NDRG1, which in turn blocks the pathway (Fig. 2).

Prostate cancer cell lines have been other sources for identification of the function of NDRG1. A chemical genetic screening method for identification of substrates for the oncogenic serine/threonine kinase PIM1 has led to identification of NDRG1 as an important substrate for this kinase in prostate cancer cells. PIM1 can phosphorylate pS330 of NDRG1, leading to reduction of stability NDRG1, its nuclear localization, and interaction with androgen receptor (AR), which consequently enhances cell migration and invasion [13]. Another study in prostate cancer has shown that N-cadherin induces expression of c-Jun and inhibits expression of NDRG1 to increase invasive properties and migratory potential of prostate cancer cells via affecting epithelial to mesenchymal transition (EMT). Moreover, c-Jun, AR, and DNMT1 establish a complex in the TPA response elements region of the NDRG1 promoter, which inhibits transcription of NDRG1 via induction of hypermethylation of DNA [14]. Two other studies have revealed a tumor suppressor role for NDRG1 in prostate cancer in association with the role of this protein in modulation of AR activity. First, NDRG1 has been shown to directly regulate AR signaling in this type of cancer [15]. Second, the impact of MLL5α on activation of AR/NDRG1 signaling has been found to result in the suppression of prostate cancer progression [16]. Table 1 summarizes the results of studies on the role of NDRG1 in different cancer cell lines.

**Table 1:** Role of NDRG1 in cancer cell lines

Tumor type	Interactions/regulatory mechanisms	Cell line	Function	References
Breast cancer (TNBC)	HJURP/YAP1 axis	T47D, ZR-75–1, BT549, HCC1937, BCAP-37, MCF-7, HS578T, SK-BR-3 and MDA-MB-231	↑ NDRG1: ↑ tumor cell proliferation YAP1 can induce transcription of NDRG1 through binding with its promoter	[4]
Breast cancer (TNBC)	mitochondria	MDA-MB-231 and SUM-159	Δ NDRG1: ↓ tumor cell proliferation	[5]
Breast cancer	Neutral lipid metabolism	SKBR3, MCF7, HCC1569, BT474, MDA-MB-231, MDA-MB-468 and HEK293T	Δ NDRG1: ↓ tumor cell proliferation	[6]
Breast cancer (PR ±)	AP-1/NDRG1 axis, kinase gene SGK1	T47D, BT474, MDA-MB-231, ZR-75–1,MCF7 and 184A1	↑ NDRG1: ↑tumor cell proliferation	[7]
Breast cancer	lncRNA NDRG1-OT1	MCF-7 and HEK293T	NDRG1-OT1: ↓NDRG1 promoter activities	[8]
Breast cancer	NDRG1-OT1_v4	MCF-7, SKBR3, MDAMB-231, ZR75-30, MDAMB-361 and MDAMB-453 and MCF-10A	NDRG1-OT1_v4 inhibits expression of NDRG1 at mRNA and protein levels NDRG1-OT1_v4: destabilizes NDRG1	[17]
Breast cancer	aryl hydrocarbon receptor (AHR)	MCF-7	↑↑AHR: ↑cell proliferation and migration via up-regulation of NDRG1 ↑ NDRG1: ↑tumor cell proliferation	[18]
Breast cancer	miR-769-3p	MCF-7	miR-769-3p inhibits the expression of NDRG1 ↑↑ miR-769-3p: ↓ cell proliferation and ↑ apoptosis	[19]
Breast cancer	methylation	MDA-MB-231 and T47D	↓NDRG1: ↑ tumor cell invasion	[9]
Breast cancer	β-casein	SK-BR-3, MDA-MB-231, T47D and MCF-7	Correlation between endogenous expression levels of NDRG1 and differentiation status	[2]
Prostate cancer	YWHAZ	–	↑NDRG1: ↑ tumor cell proliferation	[20]
Prostate cancer	PIM1	LNCaP-95, LNCaP-abl, LAPC-4, C4–2, 293 T, VCaP and 22Rv1	NDRG1 phosphorylation: ↑ cell migration and invasion	[13]
Prostate cancer	N-cadherin/c-Jun/NDRG1 axis	LNCaP and PC3	↓NDRG1: ↑ tumor cell invasion and migration	[14]
Prostate cancer	androgen receptor signaling	LNCaP, C4-2B, and 22Rv1	↓NDRG1: ↑ tumor cell growth and metastasis NDRG1 suppresses AR activation	[15]
Prostate cancer	MLL5α, AR/NDRG1 signaling	LNCaP, C4-2, 22RV1, PC3 and 293 T	↓NDRG1: ↑ tumor cell invasion and migration MLL5α suppresses cancer progression by inducing AR/NDRG1 signaling	[16]
Prostate Cancer	E6AP-NDRG1 axis	DU145, PC3, LNCaP and BPH	↑↑E6AP: ↓NDRG1	[21]
	methylation of CpG islands of NDRG1 promoter	LNCap, PC-3, DU145, 22Rv1 and RWPE-1	↓NDRG1: ↑ tumor cell proliferation and invasion	[22]
Prostate cancer	Proteolytic cleavage	DU145, PC3, LNCaP, PC3MM/Tet-Flag-Drg-1 and PrECs	↓NDRG1: ↑ tumor cell growth Cleavage of NDRG1 results in loss of tumor suppressor activity of this protein	[23]
Prostate cancer	AKT, TGF-β and ERK pathways	PrECs, DU145 and PC-3	Dp44mT: ↑ NDRG1 and PTEN expression Δ NDRG1: ↑phosphorylation of AKT, ERK1/2 and SMAD2L and ↓ PTEN level	[24]
Prostate cancer	miR-182	LNCap, PC-3, DU145, 22Rv1 and RWPE-1	miR-182: ↓NDRG1 ↓NDRG1: ↑ tumor cell proliferation	[25]
Gastric cancer	DNMT family (DNA methylation)	SGC7901, MKN45,GES1	DNA methylation: ↓NDRG1 expression ↓NDRG1: ↑ tumor cell proliferation	[26]
Gastric cancer	DNA methylation	-	DNA methylation: ↓NDRG1 expression ↓NDRG1: ↑ tumor cell invasion and migration	[27]
Gastric cancer	miR-223-3p/NDRG1 axis, Hsa_circ_0003159	GES-1, NUGC-3, AGS, HS-746 T and N87	Hsa_circ_0003159 suppresses proliferation, migration and invasion but promotes apoptosis by decreasing miR-223-3p and increasing NDRG1 ↓NDRG1: ↑ tumor cell growth	[28]
Gastric carcinoma	Several cellular signaling pathways	–	↓NDRG1: ↑ tumor aggressiveness	[29]
Gastric cancer	MMP-9	SGC7901	Δ NDRG1: ↑ cell proliferation and invasion	[30]
Colorectal cancer	CLDN2/ZO1/ZONAB-NDRG1 axis	Lovo, SW480, SK-CO15, HCT116, LIM1215, SW620, Caco2, HT29, SW480, NCM460 and HEK293T	CLDN2 depletion significantly promotes NDRG1 transcription, leading to termination of tumor growth and metastasis	[31]
Colorectal cancer	CDC42	HCT116, RKO	Δ NDRG1: ↑ dissemination of CRC cells ↓NDRG1: ↑formation of filopodia and invasiveness of cancer	[32]
Colorectal cancer	EGFR trafficking	RKO and HCT116	NDRG1 enhances the sensitivity colorectal cancer to CTX NDRG1 can increase CTX activity in metastatic cancer	[33]
Colorectal cancer	MORC2	HT‐29, SW‐480, SW‐620 and HEK‐293	MORC2 downregulates NDRG1 mRNA, protein levels, and decreases its promoter activity	[34]
Colorectal cancer	caveolin-1 ubiquitylation	HT29, SW480 and SW1116	↓NDRG1: ↑ EMT, migration and invasion NDRG1 decreases expression of caveolin-1 protein via enhancing its ubiquitylation and consequent degradation	[35]
Colorectal cancer	nuclear β-catenin and CD44	HT29 and HCT116	The anti-metastatic activity of NDRG1 in CRC is mediated via decreasing expression of nuclear β-catenin Δ NDRG1: ↑tumorigenic ability and stem cell-like properties	[36]
Rectal cancer	Genes inducing resistance to ionizing radiation	SNU-61, SNU-283, SNU-503, SNU-977, SNU-977R80Gy, SNU-1411, and SNU-1411R80Gy	NDRG1 levels were found to be increased in radio-resistant cell lines	[37]
Osteosarcoma	PI3K/AKT pathway, miR-96-5p	MG63,U2OS, HOS, 143B and hFOB 1.19	↑ LncRNA NDRG1: ↑ tumor cell proliferation and migration	[38]
Osteosarcoma	mitochondrial function and CSCs differentiation	hFOB, U2OS, and MG63	↓NDRG1: ↓ cell viability, invasion ability and ↑ cell apoptosis increased Δ NDRG1: promotes the CSCs differentiation and decreases cancer progression	[39]
Osteosarcoma	HER4	Saos2, MCF-7, CCHO and MG63.2	↑↑NDRG1: ↑ cell growth and ↓ apoptosis Δ HER4: ↓ cell growth and tumorigenesis, and ↑ cell senescence and apoptosis and ↑chemosensitivity	[40]
Ovarian cancer	CD105	OVCAR3 (OC3), PTX-resistant OC3/TAX300	Δ CD105: ↑ NDRG1 expression ↓NDRG1: ↑ tumor cell proliferation	[41]
Cervical cancer	ANXA2-NDRG1-STAT1	CaSki and C-4I	↓ANXA2 and NDRG1, and ↑ STAT1 predict sensitivity of patients to concomitant CRT	[42]
Cervical and ovarian cancer	LPXN, DDR2, COL6A1, IL6, IL8, FYN, PTP4A3, PAPPA, ETV5, CYGB and CLCA2	CaSki and HO-8910PM	Δ NDRG1: ↑ tumor cell adhesion, migration and invasion activities without affecting cell proliferation	[43]
Endometrial carcinoma	PTEN	–	↑↑NDRG1 and ↓PTEN: ↑tumor cell proliferation	[44]
Lung cancer	ATF3	Human bronchial epithelial (HBE) cells, lung carcinoma cell lines	↑↑NDRG1: ↓ cisplatin-induced cytotoxicity in lung cancer A549 cells ↑↑NDRG1: ↓ATF3	[45]
Lung cancer	HIF-1α	A549	↑↑NDRG1: ↑ tumor cell proliferation and ↓apoptosis HIF-1α binds to NDRG1 promoter and activates its expression	[46]
Lung adenocarcinoma	Digoxin, HIF-1α	A549	↑↑NDRG1: ↑tumor cell proliferation Hypoxia-induced up-regulation of VEGF, NDRG1, and HIF-1α is suppressed by digoxin	[47]
Non-small-cell lung cancer	NDRG1/Cap43/Drg-1 axis	A549, PC9, 11_18, LK87, LC-1, QG56, LC-Sq-1, and RERF-LC-AI	Δ NDRG1/Cap43: ↓ tumor growth and angiogenesis	[48]
Bladder cancer	epithelial-mesenchymal transition (EMT)	5637, T24 and UMUC3	↑↑NDRG1: ↑cell proliferation, migration, invasion and ↓apoptotic cell numbers Δ NDRG1: ↓ cell proliferation, migration, invasion and ↑apoptotic cell numbers	[49]
Hepatocellular carcinoma	LINC00844	HCCLM9, SMMC-7721, SK-Hep1, and HepG2	↑NDRG1: ↑ tumor cell proliferation ↑↑ LINC00844: proliferation, migration, and invasion LINC00844 can suppress expression of NDRG1	[50]
Hepatocellular Carcinoma	miR-188-3p and miR-133b	HepG2	↑miR-188-3p and miR-133b: ↓ NRDG1 expression and ↓ cell growth and cell migration	[51]
Hepatocellular carcinoma	LINC01419	SMMC7721 and SK-Hep1	↑ LINC01419: ↑ NDRG1 promoter activity and cell proliferation, migration and invasion	[52]
Esophageal squamous cell carcinoma	GC-GR pathway, GR /Sgk1/NDRG1 axis	–	↑activity of the GC-GR pathway with↑ induction of Sgk1 and NDRG1: tumor progression and development of chemoresistance	[53]
Nasopharyngeal cancer	Smad2 signaling	5–8F and 5–8F-LN	ΔNDRG1: ↑ tumor cell proliferation, migration, and invasion and induced EMT ↓NDRG1: ↑ tumor cell proliferation and metastasis	[54]
Pancreatic cancer	Wnt/tenascin C	AsPC-1, PANC-1, and MIAPaCa-2	NDRG1 inhibits Wnt/TnC (antioncogenic activity) ↓NDRG1: ↑ tumor cell proliferation	[55]
Pancreatic Cancer	Histone Deacetylase inhibitor	Capan-1 and PANC-1	↓NDRG1: ↑ tumor cell proliferation and invasion	[56]
Clear cell renal cell carcinoma	HIF-1/2α	786‐O, Caki‐1, RCC4/EV and RCC4/VHL	↓NDRG1: ↑ tumor cell proliferation, metastasis and invasion	[57]
Kaposi’s sarcoma-associated herpesvirus	PCNA	MM, KMM, SLK, iSLK.RGB, iSLK.LANAstop, and HEK293T, BCBL1, JSC1, BC3, DG75, Raji, Loukes, Ramous, HUVECs	↑NDRG1: ↑ tumor cell proliferation ΔNDRG1: ↓ viral genome copy number in tumor cells	[58]

## Animal studies

The bulk of evidence from animal studies has indicated a tumor suppressor role for NDRG1 in colorectal, gastric, nasopharyngeal and renal cell cancers (Table 2). However, in osteosarcoma models, up-regulation of NDRG1 has been associated with higher rate of tumor growth [40]. Similarly, in non-small cell carcinoma, NDRG1 silencing has attenuated tumor growth and reduced angiogenesis [48].

**Table 2:** Animal studies on the role of NDRG1

Tumor type	Results	References
Colorectal cancer	ΔNDRG1: ↑tumor growth and liver metastasis	[31]
Colorectal cancer	ΔNDRG1: ↑invasion and metastasis	[32]
Colorectal cancer	ΔNDRG1: ↑tumor growth and tumor weight	[33]
Colorectal cancer	ΔNDRG1: ↑tumor growth and lung metastasis	[34]
Colorectal cancer	ΔNDRG1: ↑invasion and metastasis	[35]
Colorectal cancer	ΔNDRG1: ↑tumor growth	[36]
Breast cancer (TNBC)	ΔYAP1(interaction with NDRG1): ↓tumor growth ↑↑ NDRG1: ↑ cell growth	[4]
Breast cancer	NDRG1 was co-expressed with and β-casein or MFP Up-regulation of NDRG1 resulted in the expansion of the differentiated area	[2]
Gastric cancer	ΔNDRG1: ↑tumor weight and tumor volume	[28]
Nasopharyngeal cancer	ΔNDRG1: ↑cell proliferation	[54]
Non-small-cell lung cancer	ΔNDRG1: ↓tumor growth, tumor volume and angiogenesis	[48]
Osteosarcoma	Δ LncRNA NDRG1: ↓Tumor mass and volume and lung metastasis	[38]
Osteosarcoma	ΔHER4 (interaction counterpart of NDRG1): ↓ tumor growth ↑↑NDRG1: ↑ tumor growth	[40]
Prostate cancer	↓ NDRG1: ↑ tumor growth Δ N-cadherin (interaction counterpart of NDRG1): ↓invasion and metastasis	[14]
Prostate cancer	↑↑ MLL5α (interaction counterpart of NDRG1): ↓tumor growth	[16]
Prostate cancer	Δ E6AP (interaction counterpart of NDRG1): ↓tumor growth	[21]
Clear cell renal cell carcinoma	Δ NDRG1: ↑tumor growth, ↑tumor volume and lung metastasis	[57]

## Studies in clinical samples

NDRG1 has been suggested as a prognostic marker in patients with inflammatory breast cancer. Based on the results of univariate analyses, expression level of NDRG1, tumor grade, clinical stage and estrogen receptor (ER) status have been associated with overall and disease-free survival times. Patient with over-expression of NDRG1 have exhibited poor survival times. Particularly, those having over-expression of NDRG1 and ER-negative status have been shown to have worse outcome [59].

A meta-analysis of NDRG1 levels in numerous publicly available databases has shown correlation between NDRG1 up-regulation and expression of glycolytic and hypoxia-associated genes. Moreover, over-expression of NDRG1 has been associated with enhancement of metastasis and increase in patients' mortality [6]. Moreover, another study has revealed methylation of the NDRG1 promoter in about one third of primary breast cancer specimens. Moreover, methylation of promoter of this gene has been correlated with the TNM stage, metastasis, lymph node involvement, moderate and poor histological grade in these patients [9].

Proteomic analysis in human prostate cancer and benign prostatic hyperplasia samples have revealed association between over-expression of YWHAZ and NDRG1 and poor prognosis based on Gleason scores. In fact, YWHAZ and NDRG1 expression levels could define two groups of prostate cancer patients with high and intermediate risks of mortality. Based on the multivariable analyses, expression levels of these genes predict prognosis in an independent manner from Gleason scores [20].

Expression of NDRG1 has been found to be down-regulated in gastric cancer samples. Notably, its expression has been negatively correlated with the expression levels of DNMT1, DNMT3A and DNMT3B. Consistent with this finding, DNA methylation status of NDRG1 has been positively correlated to DNMT family. NDRG1 levels have been inversely correlated with invasion depth. However, levels of NDRG1 and DNMT1 have not been associated with prognosis of gastric cancer [26]. The latter finding is in contrast with another study in gastric cancer which has reported association between down-regulation of NDRG1 and poor clinical outcome [27].

Studies in other types of cancers have also revealed dysregulation of protein or mRNA levels of NDRG1 and associations between this abnormal expression pattern and malignant behavior of cancer as reflected in survival time of patients (Table 3).

**Table 3:** Expression studies in clinical samples on the role of NDRG1

Tumor type	Samples	Expression (tumor vs. normal)	Kaplan–Meier analysis (impact of NDRG1 dysregulation)	Univariate/multivariate cox regression	Association of dysregulation of NDRG1 with clinical data	References
Breast cancer (TNBC)		Up	Shorter OS	–	–	[4]
Breast cancer (TNBC)	TCGA database (963 patients)	Up	Shorter OS	–	–	[5]
Breast cancer (IBC)	64 patients	Up	Shorter OS and DFS	NDRG1 was an independent prognostic factor for OS and DFS	Negative HER2 status	[59]
Breast cancer	TCGA database	Up	Shorter OS	–	–	[6]
Breast cancer	389 patients	Down	–	–	NDRG1 promoter methylation was correlated with TNM at stage III/IV, metastasis, lymph invasion, moderate and poor histological grade	[9]
Breast cancer	45 patients	Down	–	–	Differentiation status	[2]
Bladder cancer	100 patients	Up	Shorter OS	NDRG1 expression was an independent prognostic factor	Lymph node metastasis, TNM stage	[49]
Cervical cancer	40 patients		–	–		[42]
Colorectal cancer	104 CRC tumors and 85 adjacent normal mucosa	Down	Shorter OS	–	–	[31]
Colorectal cancer	86 PTN	Down	–	–	Advanced T stage	[32]
Colorectal cancer	65 patients	Down	Poor OS	–	–	[33]
Colorectal cancer	64 PTN	Down	–	–	–	[35]
Colorectal cancer	116 patient	Down	–	–	Positive lymph node metastasis	[36]
Colorectal cancer	119 CRC tissues and 36 Non-tumor colon tissues	Down	Shorter OS	–	Lymph node metastasis and poor pTNM stage	[34]
Endometrial carcinoma	103 patient	Up	–	–	–	[44]
Esophageal squamous cell carcinoma	98 patient	Up	Shorter OS and DFS	Univariate analysis showed association between patients' survival and expression of NDRG1	pT, pStage, and lymphovascular invasion	[53]
Gastric cancer	34 PTN	Down	Shorter OS	–	NDRG1 expression was negatively associated with tumor size, depth of invasion, lymph node metastasis, lymphatic invasion and differentiation	[27]
Gastric cancer	TCGA database 407 patients	Down	–	–	Invasion depth	[26]
Gastric cancer	101 patients	Down	–	–	Degree of tumor cell differentiation, invasion depth, lymph node metastasis and TNM stage	[30]
Gastric cancer	228 patients	Down	Shorter OS	Univariate Cox: VM, HER2, tumor size, TNM stage, lymphatic metastasis, distant metastasis and metastasis and recurrence Multivariate Cox: Only HER2, metastasis and recurrence were independent risk factors in gastric cancer	Tumor histological differentiation, TNM, Lauren type, lymph node metastasis, distant metastasis, recurrence and metastasis, and HER2 expression	[29]
Gastric cancer	55 PTN	Down	–	–	–	[28]
Hepatocellular carcinoma	40 PTN	Up	–	–	Histological grade	[50]
Hepatocellular carcinoma	43 PTN	Up	–	–	–	[51]
Nasopharyngeal cancer	83 patients	Down	Shorter OS	Lymphatic metastasis	–	[54]
Non-small-cell lung cancer	182 patients	Up	Shorter OS	–	Age and cytoplasmic NDRG1/Cap43 expression	[48]
Osteosarcoma	18 patients	Up	Shorter OS	–	Enneking stage and distant metastasis	[38]
Ovarian cancer	53 patients	Down	–	–	–	[41]
Prostate cancer	TCGA database	Up	Shorter OS	YWHAZ, NDRG1, GS, and age are independent predictors of mortality	GS, age group, and TMPRSS2-ERG fusion	[20]
Prostate cancer	60 patients	Down	Shorter OS	–	–	[14]
Prostate cancer	31 patients	Down	–	–	–	[15]
Prostate cancer	45 patients	Down	Shorter OS	–	–	[16]
Clear cell renal cell carcinoma	645 tumor samples and 260 adjacent normal tissues	Down	–	–	–	[57]

*OS* overall survival, *DFS* disease free survival, *PTN* parried tumor and non-tumoral samples

## Discussion

The role of NDRG1 has been vastly assessed in the contexts of breast, prostate, gastric and colorectal cancers. NDRG1 is an example of proteins with different roles in the carcinogenesis. These various and opposite effects can be at least partly explained by cell- or tissue-specific roles. However, experiments in a certain type of cancer have sometimes reported opposite effects for NDRG1. This is particularly true in breast and prostate cancers, two types of cancer that have been the focus of several independent studies. This discrepancy can be explained by differences in the stage, grade or other pathological features of cancer cells. Since tumor tissues are heterogeneous in terms of gene expression patterns, single cell gene expression profiling is needed to elaborate expression of NDRG1 in relation with pathological state.

The lncRNA encoded by the same region (NDRG1-OT1) has been shown to effectively influence expression of NDRG1. Aryl hydrocarbon receptor and a number of miRNAs mediate other mechanisms for regulation of expression NDRG1. Finally, methylation status of NDRG1 promoter has an established role in the regulation of its expression based on the results of studies in different tissues/ cell types.

Most importantly, the lncRNA encoded from NDRG1 locus has been shown to serve as a spong for miR-96-5p [38, 60, 61]. Since competing endogenous RNA function is an important mechanism for the regulation of expression of genes by lncRNAs, further studies should find other miRNAs that are sponged by this lncRNA to unravel additional parts from the regulatory network of NDRG1.

Expression profiles of NDRG1 in clinical samples have revealed association between dysregulation of NDRG1 and poor clinical outcomes, demonstrating the prognostic impact of this protein in the context of cancer. However, the role of this protein in the early diagnosis of cancer, particularly based on expression assays in biofluids should be discovered in future.

NDRG1 has been shown to interact with a number of transcription factors and signaling pathways such as YAP1, AP-1, aryl hydrocarbon receptor, β-casein, androgen receptor, N-cadherin/c-Jun, MLL5α, E6AP, PTEN, ATF3, HIF-1α and PI3K/AKT, Smad2, Wnt/tenascin C, TGF-β and ERK pathways. Moreover, miR-769-3p, miR-182, miR-223-3p, miR-96-5p, miR-188-3p, miR-133b, hsa_circ_0003159, LINC00844 and LINC01419 are among non-coding RNAs that interact with NDRG1. Therefore, the oncogenic versus tumor suppressor roles of NDRG1 in different tissues should be interpreted considering the extensive number of NDRG1 counterparts in each situation.

Based on the importance of NDRG1 in the pathogenesis of cancer and induction or modulation of chemo-/radio-resistance in different types of cancers, future studies are required to unravel the exact role of this protein in the tissue-specific carcinogenic processes and develop specific therapies for each type of cancer.

Taken together, the data presented above casts doubt on the previously supposed "anti-metastatic" role for NDRG1 and suggests a tissue- or cell- or stage-specific role for this protein in the carcinogenesis.

## Data Availability

The analyzed data sets generated during the study are available from the corresponding author on reasonable request.
